# Chimeric antigen receptor technology: an emerging translational immunotherapy in nonneoplastic diseases

**DOI:** 10.3389/fimmu.2026.1864256

**Published:** 2026-06-03

**Authors:** Sijie Zhou, Yuan Li, Jiulu Zhao, Wang Zhan, Cheng Zhou, Yanqiang Zou, Xiaohan Li, Jikai Cui, Jie Wu, Jiahong Xia

**Affiliations:** 1 Department of Cardiovascular Surgery, Union Hospital, Tongji Medical College, Huazhong University of Science and Technology, Wuhan, Hubei, China; 2 Center for Translational Medicine, Union Hospital, Tongji Medical College, Huazhong University of Science and Technology, Wuhan, Hubei, China; 3 Institute of Translational Medicine, Tongji Medical College, Huazhong University of Science and Technology, Wuhan, Hubei, China; 4 Hubei Key Laboratory of Biological Targeted Therapy, Union Hospital, Tongji Medical College, Huazhong University of Science and Technology, Wuhan, Hubei, China; 5 Key Laboratory of Organ Transplantation, Ministry of Education, National Health Commission (NHC) Key Laboratory of Organ Transplantation, Key Laboratory of Organ Transplantation, Chinese Academy of Medical Sciences, Wuhan, Hubei, China

**Keywords:** chimeric antigen receptor (CAR) technology, engineered immune cells, immunotherapy, nonneoplastic diseases, precision targeting

## Abstract

**Background:**

One of the most significant recent advancements in cancer immunotherapy is the development of chimeric antigen receptor (CAR) technology. More recently, this approach has been gradually modified for the research associated with various treatment-resistant nonneoplastic diseases by engineering immune cells to provide precise targeting.

**Results:**

This narrative review discusses two primary therapeutic approaches the use of CAR technology in the treatment of nonneoplastic diseases. One strategy involves the elimination of specific pathogenic cell populations. Specifically, by engineering T cells, macrophages, or natural killer (NK) cells, pathogenic cells can be eliminated in autoimmune disorders, infectious diseases, and fibrotic lesions. The second approach aims to restore immune homeostasis by using engineered regulatory T cells (Tregs) to control augmented immune effector responses. This strategy has been shown to promote transplant tolerance and has therapeutic potential for inflammatory bowel disease and type 1 diabetes. Furthermore, this review addresses major issues concerning the persistence, safety, and manufacturing accessibility of CAR cells and discusses some emerging technological approaches that could be used for focused refinements of this technology.

**Conclusions:**

The precision medicine platform for CAR technology has advanced beyond oncology by integrating targeted cell destruction with the management of immune homeostasis. As future possibilities with frontier cell engineering and interdisciplinary approaches are explored, CAR cell therapy is likely to evolve into a more adaptable, refined, and clinically viable immunotherapy technology, with its therapeutic potential expanded to a broad range of diseases.

## 1 Introduction

Recent advances in chimeric antigen receptor (CAR) technology have revolutionized immunotherapy by allowing the modification of immune cells to specifically target and eliminate desired cells. The clinical implementation of CAR cell therapy for the treatment of B-cell lineage hematologic malignancies is a significant achievement in precision medicine, as these therapies demonstrate not only potent antitumor activity but also the ability to achieve profound and durable depletion of pathogenic immune cell subsets for effective disease control (1, 2).

These advances have prompted researchers to investigate the use of CAR technology for a multitude of refractory nonneoplastic diseases, which extends its application well beyond cancer (3, 4). Preclinical and early-phase clinical studies have shown promising results for CAR T-cell therapies in the elimination of pathogenic cells in immune cell-driven disorders, infectious diseases, and fibrosis. Additionally, the immunomodulatory effects of CAR technology have shown promise in transplant immunology, inflammatory diseases, and type 1 diabetes, further highlighting the versatility and applicability of this technology in chronic disease management.

Furthermore, there are still some key knowledge gaps in this field, including the mechanisms underlying therapeutic persistence, safety and accessibility profiles, and strategies for precise immunomodulation in chronic disease settings (5). These challenges further limit the potential of translational applications. Advances in synthetic biology, gene editing, and biomaterials may provide solutions, yet systematic studies are needed to delineate the full potential and limitations of CAR cell therapies in these diseases. This review aims to summarize current progress, highlight critical challenges, and discuss interdisciplinary strategies that could advance the application of CAR cell therapies beyond oncology.

## 2 Evolution of CAR technology

The immune system defends the body by recognizing antigenic molecules. Conventional T cells recognize antigenic peptides through their T-cell receptors (TCRs), which interact with peptide fragments presented by major histocompatibility complex (MHC) molecules; however, this recognition is restricted by MHC compatibility (6, 7). CARs are engineered receptor molecules that are expressed by effector lymphocytes, most commonly T cells, to endow them with MHC-independent recognition of specific antigens and direct their cytotoxic activity (8).

**Figure 1:**
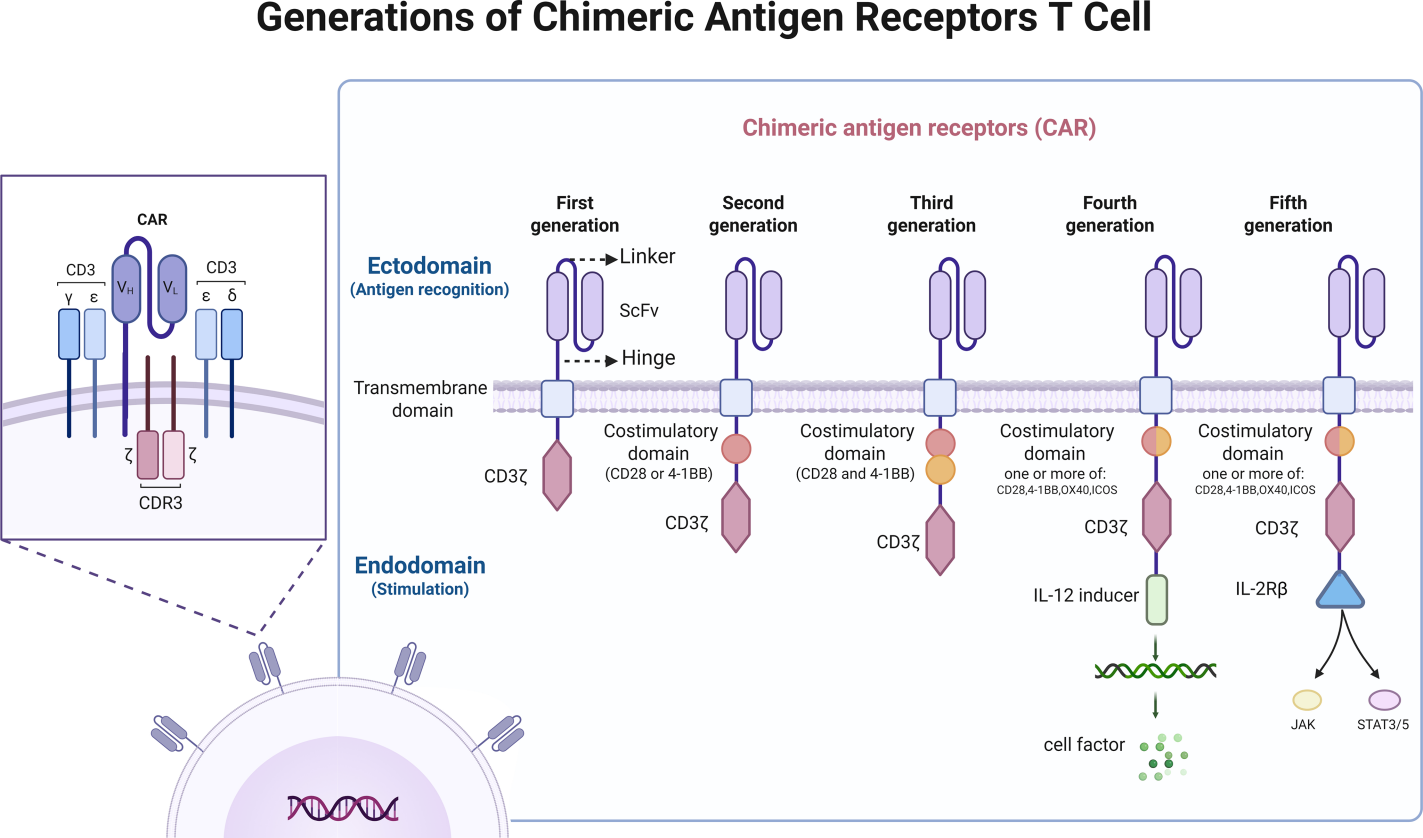
Generation of chimeric antigen receptor cells. Evolution of the CAR structure. First-generation CARs contain a CD3ζ signaling domain. Second-generation CARs have a costimulatory domain (e.g., CD28 or 4-1BB) added. Third-generation CARs combine two costimulatory domains. Fourth-generation CARs (TRUCKs) secrete cytokines. Fifth-generation CARs contain IL-2Rβ and STAT-binding domains to activate the JAK/STAT pathway. Created at https://BioRender.com.

**Figure 2:**
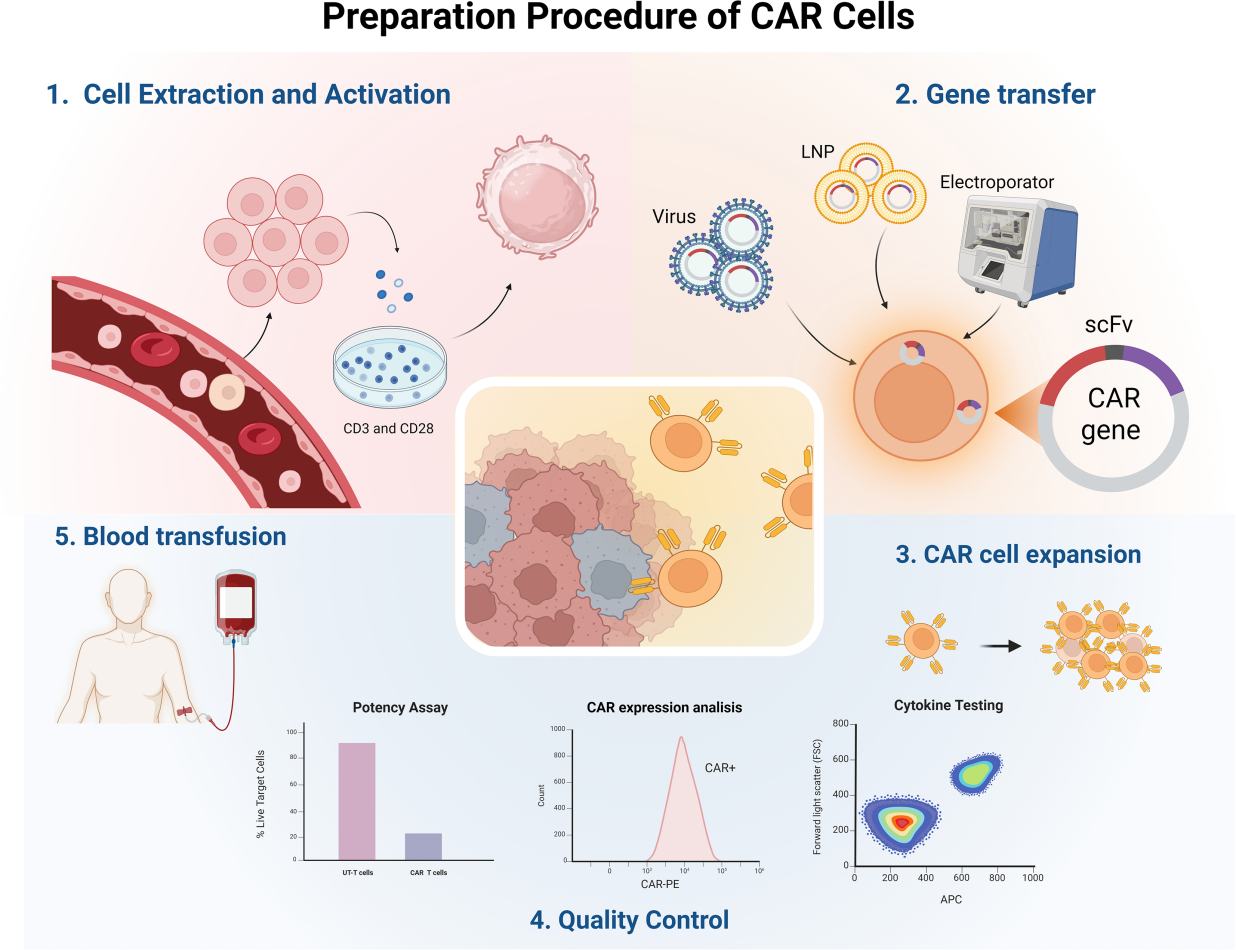
Preparation procedure for CAR cells. The preparation process for CAR cell therapy typically involves the following steps: 1. Immune cells are isolated from the patient’s peripheral blood. These cells are subsequently activated to increase their proliferation and functional activity. 2. The critical CAR gene transfer step involves the introduction of genes encoding chimeric antigen receptors into activated immune cells, which enables these cells to recognize specific antigens. This step is typically achieved through viral transduction by using lentiviral or retroviral vectors or via nonviral methods, such as liposomal transfection or mRNA/DNA electroporation. 3. The modified cells undergo *in vitro* expansion under strictly controlled conditions with supportive cytokines to obtain sufficient quantities for therapeutic use. 4. Comprehensive quality assessments of the final product are conducted to ensure product safety and efficacy, including testing for cell viability, purity, and functional potency by measuring CAR expression and cytokine release and performing cytotoxicity assays against antigen-positive targets. 5. Patients often receive lymphodepleting chemotherapy before infusion, after which these engineered cells are reinfused into the patient to perform targeted cell elimination or suppress inflammation. Each step is critical to ensure CAR cell functionality, safety, and therapeutic efficacy in patients. Created at https://BioRender.com.

The CAR is structurally organized into several functional components, including an extracellular antigen-binding domain (single-chain variable fragment, or scFv), a hinge region, a transmembrane domain, and an intracellular signaling domain, e.g., the T-cell signaling domain CD3ζ and the costimulatory molecules CD28 and 4-1BB (9). The idea of constructing a synthetic chimeric receptor, the prototype of the first-generation CAR, was originally proposed by Eshhar and colleagues in 1989 (10). These early constructs contained a fused antibody-derived binding domain coupled to CD3ζ, which enabled antigen-specific activation and cytotoxicity, but cytokine secretion was limited and unsustained (11–13). To overcome these limitations, second-generation CAR designs incorporated an additional costimulatory domain, most frequently CD28 or 4-1BB, in combination with CD3ζ signaling (14, 15). This structural modification significantly increased the proliferation and antitumor responses of engineered cells. Third-generation CAR constructs further integrate two independent costimulatory signaling domains, typically CD28 together with 4-1BB, which has been shown in preclinical studies to increase persistence and multifunctional cytokine secretion, although the clinical benefits of third-generation CARs over second-generation CARs remain inconclusive (16, 17). Fourth-generation CARs, which are commonly described as ‘armored’ CARs or TRUCKs, are engineered to secrete immune-modulating cytokines, such as IL-12, to enhance bystander immune responses (18). Fifth-generation CARs contain the intracellular domain of the interleukin-2 receptor β chain (IL-2Rβ) together with STAT3-responsive transcriptional motifs, which intervene in cellular development, survival, and memory responses by triggering downstream JAK/STAT3/5 pathways (19, 20). In summary, the ongoing core engineering optimization has aimed to improve the activation, differentiation, survival capacity, and cytotoxicity of CAR T cells (21, 22). While the extracellular antigen-binding domains and general structural components are largely conserved across CAR platforms, the intracellular signaling modules are adapted to the specific effector cell type. For instance, CAR-M, CAR-NK cells and CAR-Tregs respectively contain different signaling patterns to respectively support specialized phagocytic, cytotoxic or inhibitory functions (23, 24). Taking CAR-T cell as an example, **Figure 1** illustrates the evolutionary progression of the CAR architecture. The preparation process for CAR T-cell therapy generally involves lymphocyte collection, activation, CAR gene transfer, ex vivo expansion, quality assessment, and reinfusion (25), each step critically influencing the efficacy and safety of the final therapeutic product, as schematically presented in **Figure 2**.

The precision cell-targeting capability of CAR technology has revolutionized the treatment of hematologic malignancies. Conventional cancer treatments, including chemotherapy and radiotherapy, despite their general nonspecificity and potential damage to normal tissues, with serious side effects, are still used in patients with blood cancers. Existing studies have confirmed the significant success of applying CAR T-cell therapy to select refractory hematologic malignancies originating from B cells, with patients demonstrating unprecedentedly high response rates. These malignancies include B-cell acute lymphoblastic leukemia (ALL), multiple myeloma (MM), diffuse large B-cell lymphoma (DLBCL), and follicular lymphoma (FL) (26). Currently, 12 therapeutic products, including those targeting CD19 and BCMA, are approved for marketing by the U.S. FDA and in China. As the application of CAR technology in oncology is broadened, researchers are also turning their attention to certain nononcological diseases that are not curable by traditional therapies or are prone to recurrence. Inspired by the use of reengineered immune cells, therapeutic approaches for nonneoplastic conditions have been redefined.

## 3 Diverse applications of CAR technology

**Figure 3:**
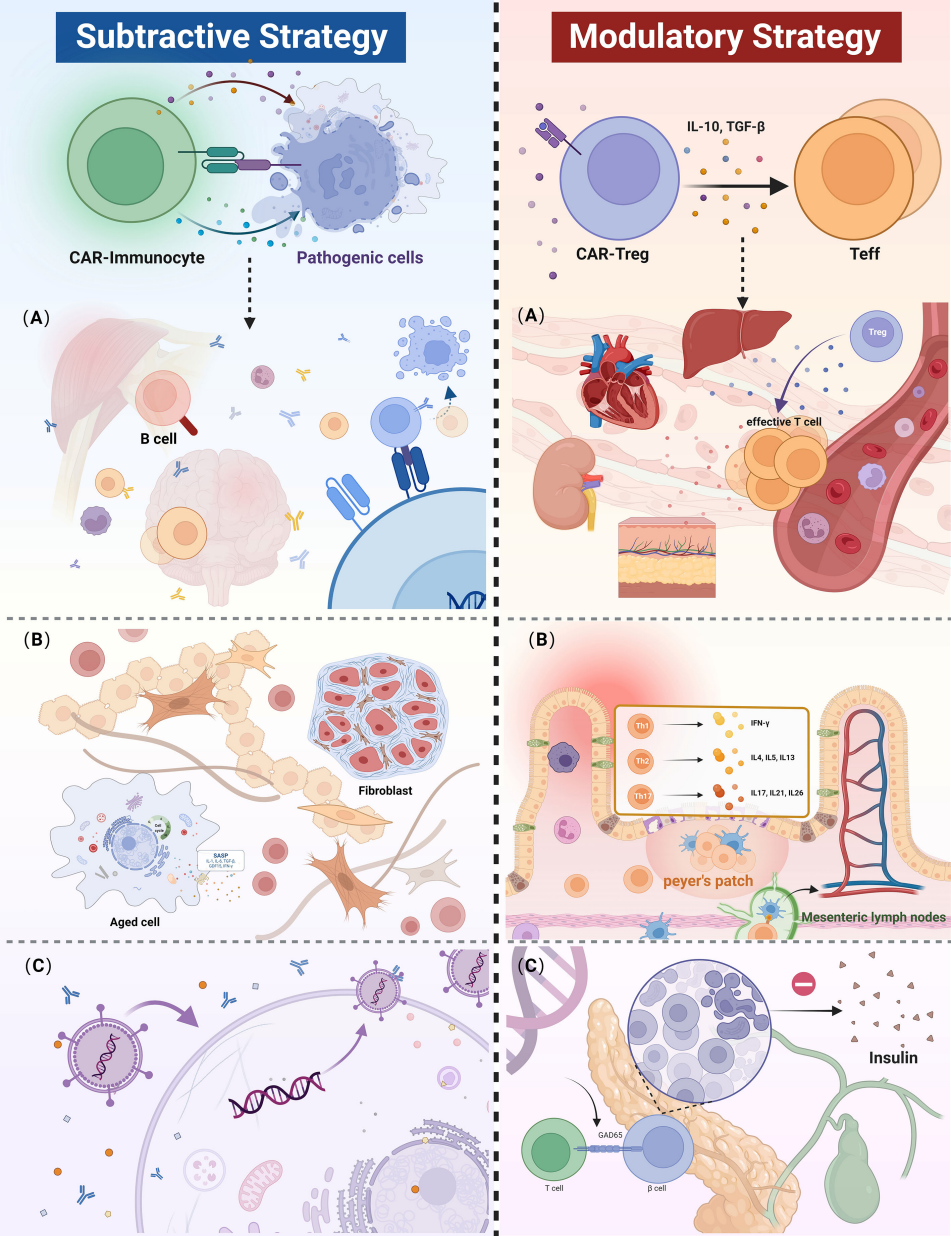
CAR cell therapeutic strategies: elimination and regulation. Left: Clearance Strategy. Effector cells are engineered to specifically target and eliminate pathogenic immune cells, infected cells, and pathological cells, thereby affecting autoimmune diseases **(A)**, fibrotic diseases **(B)**, and infectious diseases **(C)**. Right: Regulatory Strategy. By engineering Treg cells to suppress effector T-cell (Teff) activity, this approach can be used to protect tissues or organs. Its applications include inducing immune tolerance **(A)**, treating inflammatory bowel disease **(B)**, and preventing or managing type 1 diabetes **(C)**. Created at https://BioRender.com.

The programmability of CAR technology provides a flexible framework through which engineered immune cells can be redirected toward highly specific pathogenic targets while influencing, in parallel, regulatory immune populations that shape the immune environment. The combination of these two strategies within complex immune settings is driving the expansion of CAR technology beyond cancer therapy into multiple fields. The following sections elaborate on the diverse applications of these two strategies in different disease contexts, as illustrated in **Figure 3**.

### 3.1 Pathogenic cell-targeted interventions: from recognition to elimination

The most straightforward application of CAR technology is direct elimination of cells that drive disease onset and progression. This “subtractive” approach has been widely applied against multiple targets, including pathogenic immune cells, cells infected with persistent pathogens, and profibrotic cells. This has led to the application of CAR technology against numerous nonneoplastic diseases that remain difficult to cure because of the persistent presence of specific pathogenic cells.

#### 3.1.1 Clearance of immune cells

Apart from oncology, B-cell–mediated autoimmune diseases present the next most advanced and compelling area of application of CAR T-cell therapy. These diseases are generally believed to arise from dysregulated immune recognition of antigen molecules, which is triggered by genetic and environmental factors, leading to abnormal attacks and ultimately resulting in the loss of immune tolerance (27). B cells serve as core drivers of various autoimmune diseases, mediating the progression of inflammation and damage to target organs (28). B-cell–targeting therapies, exemplified by anti-CD20 monoclonal antibodies, have improved autoimmune disease treatment to a new level, but unmet clinical needs urgently necessitate more durable and thorough B-cell depletion therapy (29–33).

Given that CD19 is widely expressed in B-cell lineages, it has emerged as an attractive therapeutic target. Initial investigations in animal models of SLE provided proof-of-concept evidence indicating that CD19 CAR T cells could induce durable B-cell elimination, reduce the production of pathogenic autoantibodies, and ameliorate disease (34, 35). The functional verification of CD19 CAR T cells was subsequently expanded to clinical research in patients with systemic lupus erythematosus (SLE). In 2021, Dimitrios Mougiakakos reported the first successful clinical use of CD19 CAR T cells in the treatment of refractory SLE (36) in a patient who experienced no symptom improvement or B-cell depletion with conventional corticosteroid and B-cell–targeted biologic therapy. Following CAR T-cell infusion, the patient demonstrated marked improvements in serological markers, clinical symptoms, and SLE activity scores. Notably, no treatment-related adverse events occurred in this patient. These preliminary findings provide support for clinical application. Mackensen et al. expanded this case study to five refractory SLE patients and similarly reported that the *in vivo* proliferation of CAR T cells led to B-cell exhaustion, normalization of laboratory parameters, and improvement in clinical symptoms (37). After three months of follow-up, all treated individuals fulfilled the DORIS remission criteria and discontinued immunosuppressive agents. During subsequent follow-up, which ranged from 5 to 17 months, the patients remained relapse-free despite B-cell reconstitution. A pivotal study by Müller et al. reported clinical benefits of CD19 CAR T-cell therapy in 15 patients with refractory autoimmune diseases, including 8 with SLE, 3 with idiopathic inflammatory myopathies (IIMs), and 4 with systemic sclerosis (SSc) (38). This report confirmed the quasiabsence of serious toxicity of anti-CD19 CAR T-cell therapy and its efficacy, with all the patients demonstrating drug-free clinical remission and no further detection of autoantibodies. The encouraging results that were achieved initially in SLE patients and then confirmed across multiple autoimmune diseases have led to the rapid expansion of CD19 CAR T-cell therapy into the areas of other similar immune system disorders and hematologic disorders mediated by autoantibody formation (39–51).

Novel therapeutic strategies beyond CD19 CAR T cells, including optimizing target selection, improving cell sources, and achieving more precise immune regulation, are being actively explored. Targeting CD19 alone fails to eliminate pathogenic plasma cells with reduced or absent CD19 expression, which is the primary cause of sustained antibody production, limited response durability, and disease recurrence. Consequently, strategies targeting both CD19 and BCMA have emerged. A clinical study using dual-target CAR T-cell therapy for SLE demonstrated that all patients achieved 100% remission and maintained drug-free recovery (52). Another compelling piece of evidence from a Phase I IIT (NCT06249438) was presented at the 16th International Congress on Systemic Lupus Erythematosus. The investigators engineered novel autologous CD20/BCMA bispecific CAR T cells (C-CAR168) and demonstrated their rapid expansion, which led to complete depletion of peripheral blood B cells, T cells with low CD20 expression, and plasma cells. All four LN patients reached the 6-month efficacy assessment milestone, achieving and maintaining a systemic lupus erythematosus response index 4 (SRI4), with 3 patients meeting the criteria for remission. Building on this success in patients with SLE, Zhang et al. conducted a clinical study assessing the safety of CD19/BCMA-directed CAR T cells in patients with rheumatoid arthritis (RA) (53). The results demonstrated that this approach efficiently cleared pathogenic antibodies and rapidly alleviated clinical symptoms. A current follow-up revealed no recurrence or worsening of symptoms, with no serious adverse events reported.

Notably, all the patients utilized patient-specific autologous T cells. However, existing challenges in autologous T-cell engineering lead to failure to meet the clinical demands for standardized and large-scale manufacturing. Consequently, increasing attention has been given to the development of allogeneic T-cell–based strategies. While allogeneic rejection and GVHD have historically limited the therapeutic application of allogeneic CAR T cells, a recent study has made allogeneic clinical treatment feasible (54). Chunmei Yang and colleagues first attempted to apply allogeneic anti-CD19 CAR T-cell therapy to treat four patients with refractory SLE (55). CAR T cells, which were engineered via CRISPR/Cas9 gene editing technology, improved symptoms in all the patients, with favorable three-month follow-up outcomes. Despite the mild cytokine release syndrome (CRS) observed, allogeneic CAR T-cell therapy exhibited a favorable safety profile and demonstrable therapeutic efficacy.

The core mechanism of autologous or allogeneic CAR T-cell therapy remains the broad-spectrum elimination of B cells. While this approach has fewer adverse reactions than traditional immunosuppressants do, researchers recognize that many autoimmune diseases require more precise immune regulation. This need has spurred the development of technologies aimed at selectively eliminating autoreactive B-cell populations. Zhang et al. designed CAR T cells capable of recognizing FITC-conjugated citrullinated peptide epitopes (56). The study demonstrated the dose-dependent ability of these cells to specifically target and eliminate both antigen–peptide-immunized hybridoma cells and autoreactive B cells. Oh et al. focused on myasthenia gravis (MG) therapy and developed CAR T cells capable of precisely targeting MuSK autoantibody-positive B cells in an MG model, which significantly decreased circulating anti-MuSK IgG titers without substantially altering total B-cell counts (57). Similarly, in their 2025 follow-up study, CAR T cells expressing the AChRα subunit (A210) were engineered to specifically eliminate AChR autoreactive B cells (58). However, *in vivo* CAR stability was insufficient and requires further optimization in subsequent studies. Additionally, studies confirming that PD-1–targeted CAR NK cells can specifically eliminate overactivated immune cells open new avenues for treating autoimmune diseases that require long-term intervention (59).

In light of the above findings, pinpointing pathogenic B cells has revolutionized therapeutic strategies for B-cell–driven diseases, particularly autoimmune disorders. This core principle of specifically targeting key immune cells has advanced research into the area of eosinophil-dominant allergic diseases. For example, the essence of allergic asthma lies in the body’s production of a type 2 immune response that is triggered by reactive allergens (60). Following exposure to these reactogens, effector cells massively secrete cytokines that constitute the upstream signals of the inflammatory cascade, thereby driving chemotaxis centered on eosinophils as the core effector cells (61–64).

Monoclonal antibody therapy targeting type 2 cytokines revealed that T2 inflammation and eosinophilia constitute an intervenable pathogenic axis (65–67). Chen et al. engineered IL-5–CD28–CD3ζ receptors to confer an eosinophil-targeting ability to T cells (68). The modified cells demonstrated precise targeting and killing eosinophils, ameliorating lasting airway inflammation for up to three months, and alleviating asthma in mouse trials. Jin et al. chose IL-5 as a viable target and knocked out Bcor (which encodes a BCL-6 core silencing factor) and Zc3h12a (which encodes ZC3H12A, an endonuclease) in T cells to produce CAR T cells that were able to persist long term and kill IL-5α+ cells dominated by eosinophils (5TIF cells) (69). They also further engineered these cells to inhibit IL-4 and IL-13 signaling, thus developing 5TIF4 cells. Both *in vivo* and *in vitro* experiments demonstrated that these cells underwent collective expansion and targeted eosinophils for elimination, with 5TIF4 cells exhibiting enhanced anti-inflammatory capabilities. Finally, the humanized 5TIF4 cells constructed by the team demonstrated equivalent functionality in NSG mice.

These studies suggest that CAR cell therapy with the strategy of clearing immune cells holds promise for shifting the focus from chronic management of immune cell-driven diseases to a potential curative approach.

#### 3.1.2 Clearance of infectious cells

While CAR T cells have recently shown notable effectiveness against pathogenic immune cells in autoimmune disorders, their earliest applications were in AIDS, providing a conceptual basis for pathogen-targeted interventions in other infectious diseases. In recent decades, noticeable advances have been made in preventing and controlling infectious diseases caused by pathogens. However, the threat to global public health persists. It is currently projected that infectious diseases will continue to cause mortality at the current annual rate of 13–15 million people until at least 2030 (70). Although the innate and adaptive immune systems cooperate to prevent pathogen invasion or eliminate pathogens, various pathogens can still mediate chronic diseases through immune evasion or by inducing effector cell dysfunction (71, 72). Therefore, for the treatment of infectious diseases, CAR technology, which endows effector cells with enhanced targeting and cellular activity, offers a novel pathway to completely eliminate pathogens and restore immune surveillance.

Acquired immunodeficiency syndrome (AIDS) is a chronic life-threatening condition resulting from infection with human immunodeficiency virus (HIV), a pathogen that progressively compromises the host immune system (73). As early as 1997, Yang et al. conducted the first proof-of-concept study on the use of CAR technology against AIDS (74). They engineered T-cell receptors with human CD4- or HIV-1–specific Ig sequences and transduced them into CD8+ CTLs, enabling these cells to recognize infected cells that expressed HIV-1 gp120 on their surface. *In vitro* studies demonstrated the ability of these cells to lyse target cells and suppress HIV-1 replication. More noteworthy, the introduction of broad neutralizing antibody (BNAb) targets and CCR5 enables CAR T cells to stably recognize HIV. In addition to more potent suppression of HIV replication, these cells acquire self-protective capabilities, such as resistance to HIV infection (75, 76). The exploration of multifunctional CARs subsequently led to initial successes. Anthony-Gonda engineered anti-HIV DuoCAR T cells to address epitope loss caused by the high mutation rate of HIV. In a mouse model of HIV infection, which was established by injecting HIV-infected PBMCs, these cells demonstrated highly efficient and specific killing of HIV-infected cells (77). Moreover, the Mao team developed M10 cells expressing endogenous broadly neutralizing antibodies (BNAbs) and the follicular homing receptor CXCR5, which endowed the cells with cytotoxic, virus-neutralizing, and follicular homing capabilities. Surprisingly, these cells preliminarily demonstrated efficacy and safety in a small Phase I early-stage clinical trial (78). Moreover, the cells demonstrated promising therapeutic potential during the exploratory phase of clinical research. Moreover, CAR therapy has presented remarkable therapeutic possibilities during the exploratory phase of clinical trials and NK cell remodeling (79–82).

Strategies using engineered T cells have likewise been applied to chronic hepatitis B. Beginning in 2003, Bohne’s research group was the first to investigate the possible use of CAR cell therapy against chronic hepatitis (83). They created CAR T cells directed toward the HBV surface protein and demonstrated that the engineered T cells could distinguish HBsAg-positive hepatocytes and lyse cells that were actively replicating HBV. More importantly, T cells designed to target cccDNA allow for more complete viral elimination rather than just suppression. The safety and antiviral efficacy of CAR T cells were verified in humanized mice with viral infection. The antiviral efficacy was proven by decreases in HBsAg levels and viral load (84–86). On the basis of this evidence, the Klopp group also improved the CAR design to include safety switches of inducible cysteine protease 9 (iC9) or herpes simplex virus thymidine kinase (HSV-TK) (87). These cells retained the ability to specifically and actively kill target cells but could also undergo rapid drug-induced apoptosis. This strategy of controllable management reduces the number of infused T cells and, in turn, reduces the risks of treatment-related toxicity and the occurrence of CRS or on-target/off-tumor toxicity.

Currently, the tactics used in targeting infected cells are being applied to new enemy pathogens. Dectin-1 is among the host molecules that are involved in antifungal immunity and is critical in the immune response against species of Aspergillus. Previous studies have shown that dectin-1 contributes to immune defenses when the invading pathogen is Aspergillus fumigatus (88). Building upon this evidence, Kumaresan’s team designed a dectin-1–based chimeric antigen receptor (D-CAR), which combined this pattern recognition receptor with a fungal cell wall component, specifically β-glucan (89). T cells that express this receptor exclusively recognize species of Aspergillus and have a strong antifungal response. Thus, researchers suggest that CAR T-cell technology may be applied in antifungal immunotherapy. Additional target molecules may be identified to refine pattern recognition targeting and develop a more focused molecular targeting approach. This was the case when the conserved protein antigenic domain (AB90-E8) contained in the hyphae of Aspergillus fumigatus was identified and when a glucosidase (GXM), which is a critical virulence component of Cryptococcus, was recognized (90, 91).

Comparable concepts are also beginning to appear in antibacterial research, particularly in studies addressing implant-associated infections caused by *Staphylococcus aureus*. Considering the central role of macrophages in antibacterial immunity, Li et al. employed a surface nanodelivery strategy for implants to locally induce CAR M cells that are capable of surface-targeting *Staphylococcus aureus* surface protein A (SasA) and caspase-11 shRNA (92–95). These cells maintain potent bactericidal capacity and enhance antimicrobial function by promoting mitochondrial recruitment, which supports ROS production. In related work, the Tang team utilized CRV peptide-modified lipid nanoparticles (CRV/LNP-RNAs) to deliver SasA-CAR mRNA and CASP11, an intracellular evasion target in MRSA, to generate transient CAR M cells (96). These cells exhibited antigen-specific phagocytosis and degradation of MRSA, while mutant strains partially evaded immune surveillance.

#### 3.1.3 Clearance of pathological cells

Here, “pathological cells” denote disease-driving cell populations that disrupt tissue homeostasis and can be selectively targeted by CAR-based therapies to restore function and reduce pathology. Organ fibrosis represents the terminal pathological outcome of various chronic injuries, commonly affecting vital organs, such as the heart, liver, and lungs. It is a widespread and insidiously fatal process; however, to date, no valid targeted therapies have emerged that can reverse or halt fibrosis progression (97, 98). Fibroblast activation protein (FAP) is recognized as a core component of fibrosis progression, and its expression is upregulated in multiple fibrotic diseases. Studies have confirmed its profibrotic role in chronic diseases (99, 100).

In 2019, Aghajanian et al. initially investigated the use of CAR T-cell therapy for cardiac fibrosis and proposed a new approach to fibrosis treatment (101). This study employed a mouse model of AngII/PE-induced hypertensive cardiac injury and fibrosis. Antigen-specific CD8+ T cells were engineered to target FAP, a marker that is highly expressed on activated cardiac fibroblasts. Compared with AngII/PE-exposed mice, those receiving FAP CAR T-cell adaptation therapy exhibited marked recovery of both systolic and diastolic cardiac functions, which was accompanied by a pronounced reduction in the myocardial fibrotic burden. Notably, the sustained *in vivo* persistence of reinfused CAR T cells may compromise physiological wound repair. On the basis of the *in vivo* CAR engineering strategy involving LNPs, transient antifibrotic transgenic CAR T cells were generated. *In vitro* studies confirmed the specific killing capacity of these cells. In mouse models of organ fibrosis, anti-FAP CAR T-cell therapy significantly reduces organ fibrosis, improves organ function, and has transient therapeutic effects (102, 103). This strategy has produced beneficial effects in experimental models of both myocardial fibrosis and liver fibrosis, paving the way for personalized therapeutic approaches.

In addition to T cells, the potent phagocytic function, superior infiltration capacity, and antigen-presenting capabilities of macrophages make them more advantageous platforms for engineered cells. Research has demonstrated that CAR Ms targeting FAP+ myofibroblasts not only reduce fibroblast numbers, intervene in the organ fibrosis process, and improve cardiac function but also have tremendous potential to overcome the time window limitations inherent in traditional I/R therapies (104, 105). However, increased apoptotic cell accumulation postmyocardial infarction (MI) overloads the phagocytic capacity of macrophages, diminishing their antifibrotic potential. To address this issue, the Liu team expanded the LNP-mediated CAR platform by designing LNPs coated with dual mRNAs encoding lysosomal cysteine protease (Lgmn) and an anti-FAP CAR (106). Upon targeted phagocytosis by macrophages, these LNPs generate fibrosis-specific CAR MΦs, with dual abilities for targeted killing and enhanced cellular clearance. This synergistic approach counteracts the complex pathological environment following myocardial infarction, thereby improving myocardial fibrosis. Similar findings were reported by Mao et al., who demonstrated the therapeutic potential of FAP-CAR Ms for liver fibrosis in a CCl_4_ injection-induced mouse model (107). Interestingly, in this study, FAP-CAR-ΔZETA BMDMs, which lacked the intracellular CD3ζ signaling domain, demonstrated better therapeutic efficacy, providing a new outlook on macrophage engineering without the use of conventional T-cell activation mechanisms.

Fibrosis and ageing are pathologically intertwined. Recent research has revolutionized fibrosis treatment by targeting a more common upstream mechanism—cellular senescence. In 1961, Hayflick and colleagues first described cellular senescence, which was subsequently defined as irreversible cell cycle arrest (108–110). The senescence-associated secretory phenotype (SASP) is among the distinctive characteristics of senescent cells. Their function is not diminished with cell cycle arrest; instead, they persistently secrete a wide array of cytokines in large quantities to trigger inflammation, leading to tissue damage and functional decline (111, 112). Clearing senescent cells has been shown to improve tissue function and restore homeostasis in age-related diseases, expanding the role of CAR T cells in long-term disease prevention (113–115).

Urokinase-type plasminogen activator receptor (uPAR) and the ligand for the activated natural killer (NK) cell receptor (NKG2D) have been confirmed as universal surface markers of senescent cells (116, 117). The Amor team (116) first demonstrated the ability of uPAR-CAR T cells to clear senescent cells and reverse age-related pathological states. In addition to improving existing metabolic dysfunction, this approach provides long-lasting protection against age-related diseases, expanding the role of CAR T cells in long-term disease prevention (118). In studies targeting NKG2DLs, CAR T cells were proven to possess antigen-specific targeting and killing capabilities, reducing the number of senescent cells and improving age-related phenotypes across multiple models (119, 120).

In summary, clearance strategies extend beyond immune cells to include fibrotic cells that disrupt tissue structure and function. The boundaries of this approach are now expanding into more fundamental biological ageing processes.

### 3.2 Homeostasis-directed interventions: from immune dysregulation to recalibration

Although CAR technology has proven effective in the depletion of pathogenic cell populations, researchers have begun investigating the possibility of directly engineering immune tolerance by designing modified Tregs to suppress effector cells; hence, their mechanisms can be applied to extend areas of influence in transplant tolerance, inflammation control, and autoimmune disease suppression. This strategy focuses on more extensive and more complete remodeling of the immune microenvironment, thereby shifting the therapeutic approach from depletion to modulation.

#### 3.2.1 Immune tolerance in transplantation

Immune cell-mediated allograft rejection following transplantation remains a critical challenge affecting graft outcomes. To date, the core goal in transplant medicine has been inducing organ-specific tolerance rather than achieving complete immunosuppression (121–123). T-cell–mediated cellular immunity serves as the primary driver of acute rejection. Mismatches between donors and recipients in terms of allogeneic nonself antigens, represented by the MHC, activate T cells through direct, indirect, or semidirect pathways, triggering intense immune rejection responses (124, 125). Recent studies have increasingly explored the use of CAR Tregs as a strategy to dampen pathogenic T-cell responses and promote immune tolerance. Given that MHC class I molecules, particularly widely distributed HLA-A2, are highly expressed across all graft cells, their mismatch represents a critical factor that is closely associated with poor transplant outcomes (126, 127).

In 2016, MacDonald et al. first engineered HLA-A2–targeted CAR Tregs (128). Upon encountering specific antigens, these cells highly express activation markers (e.g., CD25) and molecules associated with suppressive function (e.g., CTLA-4) while maintaining massive secretion of the inhibitory cytokines IL-Given the high expression of MHC class I molecules across all graft cells, particularly HLA-A2, which is widely distributed in the population and whose mismatch is closely correlated with poor transplant outcomes (126, 127).

The secretion of IL-10 and TGF-β supports the notion that HLA-CAR Tregs undergo antigen-specific activation and mediate immunosuppressive effects. Following infusion, these cells maintain a stable Treg phenotype in mice. This work highlights the fundamental evidence for the stability, safety, and efficacy of HLA-CAR Tregs. Subsequent studies have demonstrated the superior therapeutic potential of HLA-CAR Tregs (129–132). Pierini et al. engineered monoclonal antibody (mAb) CAR Tregs targeting MHC class I proteins in allogeneic transplants (133). Transient expression of this mAb strongly promotes antigen targeting to Tregs, prolonging both allogeneic graft survival and extending the lifespan of the secondary skin graft that is specifically matched to the original graft.

Currently, this immunomodulatory strategy is being employed in clinical trials to assess its feasibility. A recent and ongoing clinical study assessed the safety and tolerability of A2-CAR Tregs in living kidney transplant recipients. Eight kidney transplant patients received treatment with autologous naive Tregs (TX200) engineered with an HLA-A2–targeted CAR. The results revealed stable renal function in the treatment group, which allowed an immunosuppressant dose reduction, with an extremely low incidence and low severity of adverse events (134). Similarly, Quell Therapeutics, Ltd., is conducting the LIBERATE trial (NCT05234190) to assess the safety and therapeutic efficacy of a multimodule HLA-A2–targeted CAR Tregs (QEL-001) in a cohort of 18 liver transplant recipients with HLA-A2 mismatches. However, successful transplant immunology management extends beyond controlling host-versus-graft reactions during transplantation. Importantly, in hematopoietic stem cell transplantation, one of the most notable manifestations is graft-versus-host disease (GVHD), which results from erroneous immune recognition, leading to the immune system’s ‘reverse attack’. Previous studies have confirmed that Tregs are key mediators in the prevention of GVHD following allogeneic hematopoietic stem cell transplantation (HSCT) (135–137). Rui et al. proposed a functional CAR Treg approach to targeting non-HLA antigens (138). In inflammatory environments, the upregulation of OX40 ligand (OX40L) expression on antigen-presenting cells (APCs) induces robust T-cell responses (139). In this study, they engineered anti-OX40L CAR Tregs and demonstrated through coculture assays that OX40L CAR Tregs could antigen-specifically activate and suppress the APC-mediated activation and proliferation of T cells. Crucially, a study using a human xenograft mouse model of GVHD revealed that OX40L CAR Treg therapy improved clinical scores and prolonged survival. This study provides a novel approach for targeting activated APCs.

#### 3.2.2 Modulation of inflammatory bowel disease

The lessons learned from the use of CAR Tregs to enforce immune tolerance are now being applied to address uncontrolled inflammatory disorders (140). Inflammatory bowel disease (IBD) is a type of immune-mediated disorder affecting the intestines, and its core pathogenesis is considered to be an imbalance between inflammatory activation and suppression (141). Tregs not only modulate the intestinal immune system to suppress proinflammatory factor production but also respond to local cytokine signals to regulate inflammation development (142, 143). Thus, Tregs play complex and multifaceted central roles in maintaining immune homeostasis and driving pathological processes of IBD.

In 2008, Elinav et al. generated transgenic mice that were engineered to express Tregs targeting the model antigen 2,4,6-picric acid (TNP). *In vitro*, these cells demonstrated antigen-specific recognition and suppressive functions without costimulatory signals (144). In a mouse model of TNBS-induced colitis, adoptively transferred TNP-CAR Tregs exhibited antigen-specific migration to colonic mucosal lesions, where they suppressed T-cell function, significantly improving clinical symptoms and survival rates. To advance clinical applicability, the Elinav team further employed an *in vitro* genetic engineering strategy to enable efficient transduction and expansion of natural Tregs (nTregs). The engineered cells retained normal Treg phenotypes. Crucially, consistent with the transgenic model described above, CAR Tregs functionally suppressed Teff cells and ameliorated colitis symptoms (145). In 2012, Desreumaux et al. isolated Tregs (CATS1) from Crohn’s disease patients and engineered them into ovalbumin-specific Tregs (OVA-Tregs) (146). On the basis of the evaluation of clinical indicators and laboratory parameters, some patients demonstrated favorable tolerability and preliminary signs of clinical activity. However, this trial was not designed to establish definitive efficacy. These data demonstrate the therapeutic potential of antigen-specific Tregs. Consequently, considerable effort is now being directed toward engineering Tregs as a strategy for the management of IBD.

Given the high expression of CEA in ulcerative colitis (UC) and related cancers, Blat et al. advanced their research to identify new targets. They engineered CEA-targeted CAR Tregs and evaluated their function in a mouse model of CEA-induced colitis (147, 148). Luciferase-based monitoring revealed that CEA-CAR Tregs exhibited organ-specific homing. In a CD4+ Teff-induced CEA mouse colitis model, compared with control mice, mice treated with CEA-CAR Tregs showed no weight loss and demonstrated significantly lower colitis severity. Furthermore, they demonstrated comparable colitis suppression and significant modulation of the subsequent tumor burden in a colitis-associated carcinogenesis model (AOM/DSS model). This study provides compelling support for the therapeutic value of CAR Tregs. In 2024, Cui et al. targeted the IL-23/IL-17 inflammatory axis, a pathway recognized as a central driver of Crohn’s disease (CD), by engineering CAR Tregs directed against the disease-associated receptor interleukin-23 receptor (IL-23R) (149). They demonstrated that IL23R-CAR Tregs could specifically recognize and suppress CD-related inflammatory responses while maintaining a stable phenotype. Furthermore, in humanized mouse models, these cells demonstrated robust antigen-specific migratory capacity and could be activated *in vitro* via colon biopsy cells from patients with active CD.

Recently, research on CAR technology for parallel engineering optimization and clinical translation has advanced. Through advanced CAR engineering and multistage screening strategies, Gentibio developed GNTI-932. In preclinical models, these low-immunogenicity, gut-specific CAR-engineered regulatory T cells (CAR Tregs) preferentially localize to the gut, proliferate significantly at sites of inflammation, and improve colitis symptoms. These findings demonstrate the significant potential of tissue-specific engineered Tregs for achieving more precise and durable therapeutic effects.

#### 3.2.3 Management of type 1 diabetes

Type 1 diabetes mellitus (T1DM) is characterized by immune-mediated destruction of insulin-secreting pancreatic β cells, posing significant challenges for achieving antigen-specific tolerance (150). At present, a unique obstacle to using CAR technology for treating diabetes lies in the nature of insulin as an autoantigen. As a soluble monomeric protein, it cannot effectively bind to and activate CARs, and engineered cells failed to demonstrate efficacy in disease models early (151, 152).

Recent developments have focused on antigen peptides as targets of pathology. Spanier et al. focused on the insulin B chain peptide 10–23 (InsB:9-23) presented by the IAg7 MHC class II allele present in nonobese diabetic (NOD) mice to create islet antigen-recognizing CAR Treg clusters (153). The InsB-g7 CAR was shown to significantly promote antigen-specific proliferation and suppression. In an immunodeficient NOD mouse model, InsB-g7 CAR Tregs circumvented the induction of diabetes by BDC2.5 T cells. Notably, spontaneous autoimmune diabetes was markedly delayed in immunocompetent wild-type NOD mice that received CAR Tregs. Nonetheless, the peptide-MHC targeting technique resembles physiological T-cell recognition and poses a dilemma. While it maintains a high degree of specificity, the challenges associated with MHC genetic diversity and heterogeneity remain. Instead, Shahnawaz et al. created CAR Tregs that targeted the pancreatic beta-cell antigen glutamic acid decarboxylase (GAD65) (154). This approach involves a different, more direct form of antigen recognition and does not require MHC presentation, which means, in principle, that it could be used for a much larger and heterogeneous patient population. The initial assessment of the potential for this technique was conducted in islet coculture and *in vitro* experiments. Under these conditions, the GAD65 antigen is recognized by CAR Tregs in an MHC-independent manner, and strong cell proliferation is achieved, leading to a considerable decrease in the level of autoreactive cytotoxic T lymphocytes (CTLs). Despite these favorable conditions, the quantity of the antigen that is presented on pancreatic β cells, the quality of the target epitope, and off-target effects remain highly variable. Research on T1DM is still in preclinical stages, and available mouse models are unable to adequately mimic the complexity of human diseases, with clinical correlates in patients still lacking (155). To overcome these obstacles, reengineered Tregs that target redirection of origin epitopes of autoimmune attacks have opened avenues to possibly treat T1DM. However, there are still clinically unripe elements in this therapy that need to be explored to understand the mechanistic and translational obstacles to clinical use.

Beyond this, the rapid advancement of CAR Treg therapy has also propelled progress across multiple fields, including multiple sclerosis, Alzheimer’s disease, hemophilia, autoimmune uveitis, rheumatic diseases, and atherosclerosis (156–163). However, the clinical efficacy and pharmacodynamic outcomes of this therapy still require further validation and improvement.

Overall, the CAR Tregs approach aimed at maintaining internal balance, especially the CAR Tregs, demonstrate the potential to re-adjust the immune response and restore immune tolerance. This complements the cell clearance strategy and highlights the versatility of the CAR technology.

## 4 Challenges and breakthroughs in CAR technology development

**Figure 4:**
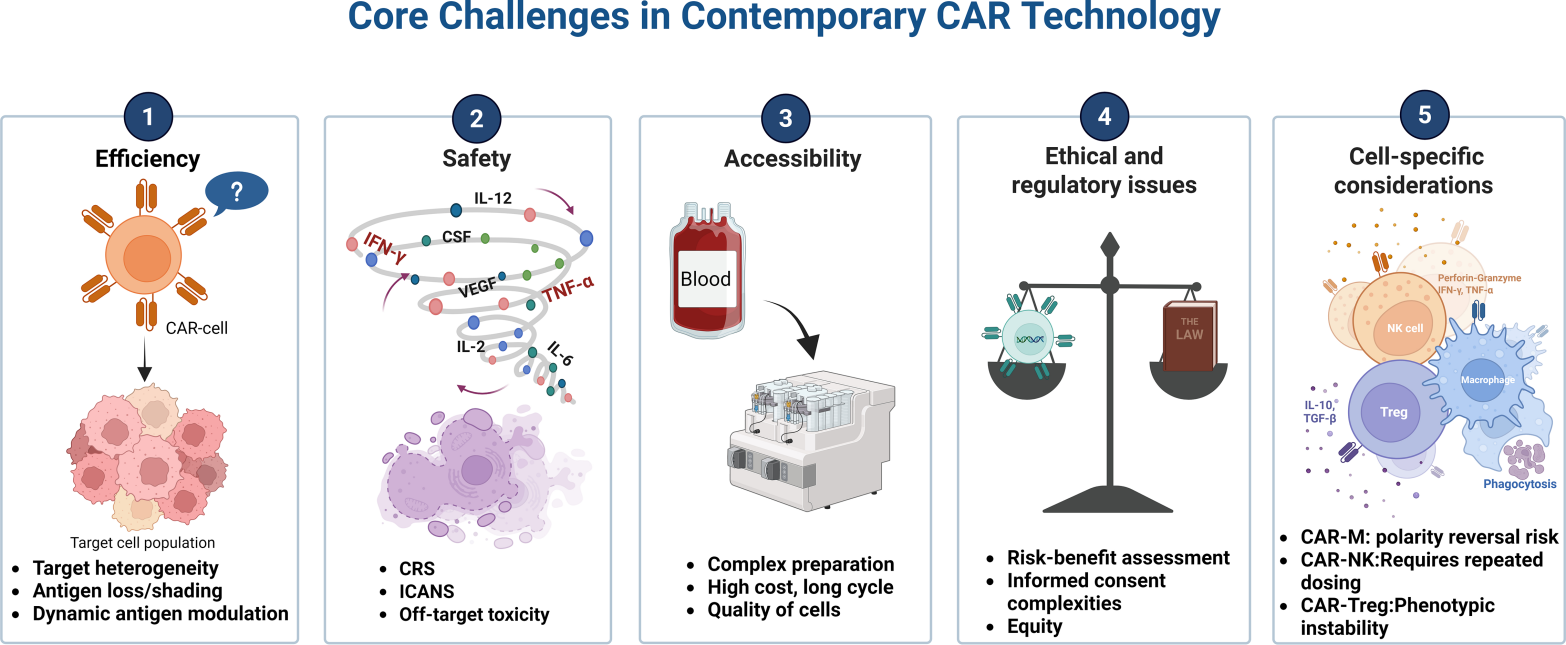
Core challenges in contemporary CAR technology. Five principal challenges in the development and clinical translation of CAR-based therapy have been encountered: 1. Efficacy: When effector cells expressing chimeric antigen receptors (CARs) encounter target cell populations, factors such as target cell heterogeneity, antigen loss or masking, and dynamic antigen regulation lead to incomplete clearance and disease recurrence, thereby limiting therapeutic efficacy. 2. Safety: The transient release of inflammatory mediators following CAR cell infusion has multiple toxic effects, including cytokine release syndrome (CRS), immune effector cell-associated neurotoxicity syndrome (ICANS), and nontargeted tissue damage. 3. Accessibility: Current virus-based autologous cell preparation processes are complex and involve intensive pretreatment procedures, high costs, lengthy production cycles, and inconsistent cell quality. 4. Ethical and regulatory issues: The widespread application of CAR cell therapies necessitates addressing multiple challenges, including defining appropriate risk–benefit thresholds for chronic nonmalignant diseases, ensuring genuine informed consent, and achieving equitable access despite limited production and follow-up capabilities. 5. Cell-specific considerations: CAR-modified immune cell populations exhibit lineage-specific advantages and risks in their interactions. CAR Ms carry a risk of polarity reversal, CAR NK cells require repeated administration because of their limited survival, and CAR Tregs pose challenges because of their phenotypic instability and insufficient control over their inhibitory effects. These challenges inform ongoing engineering strategies and clinical design considerations to maximize efficacy while minimizing adverse effects. Created at https://BioRender.com.

Over the past two to three decades of CAR technology development, encouraging outcomes in treating various refractory diseases have been demonstrated. A review of the research journey highlights key obstacles in CAR therapy development, including efficacy, safety, accessibility, ethical and regulatory concerns, and cell-specific considerations, as detailed in **Figure 4**, emphasizing both the biological and translational challenges encountered in diverse disease contexts. During the early exploratory phase, CAR technology faced numerous technical bottlenecks and challenges. These included precisely identifying and targeting tumor cells to ensure therapeutic efficacy and specificity, as well as improving the persistence and proliferation capacity in patients to sustain long-term therapeutic effects. First-generation CAR technology provides preliminary solutions for targeting, but its persistence and expansion capacity remain limited. Through the introduction of costimulatory domains and cytokine regulation, second- to fifth-generation CARs have achieved certain breakthroughs; however, persistent issues, such as recurrence and toxic reactions, remain. In recent years, process optimization and combination therapy strategies have improved therapeutic efficacy.

### 4.1 Efficacy

Disease recurrence often stems from the heterogeneity of target cells, whose antigenic mutations or masking can render CAR-modified immune cells ineffective. This is less frequently due to antigen loss in malignant tumor clones and more likely arises from stress selection and phenotypic plasticity during the immune response process. These findings indicate that target cells alter antigen expression in response to differing activation states or inflammatory signals (164). A clinical study of CD19 CAR T-cell therapy for SLE revealed that reconstituted B cells exhibited altered phenotypic and functional characteristics, with significantly reduced self-reactivity, indicating immune adaptation to target antigen expression (36). Compared with tumor cells, nonmalignant cells may be more susceptible to reversible phenotypic changes, as their target antigen expression exhibits dynamic regulatory properties rather than genetic fixity (165, 166).

**Figure 5:**
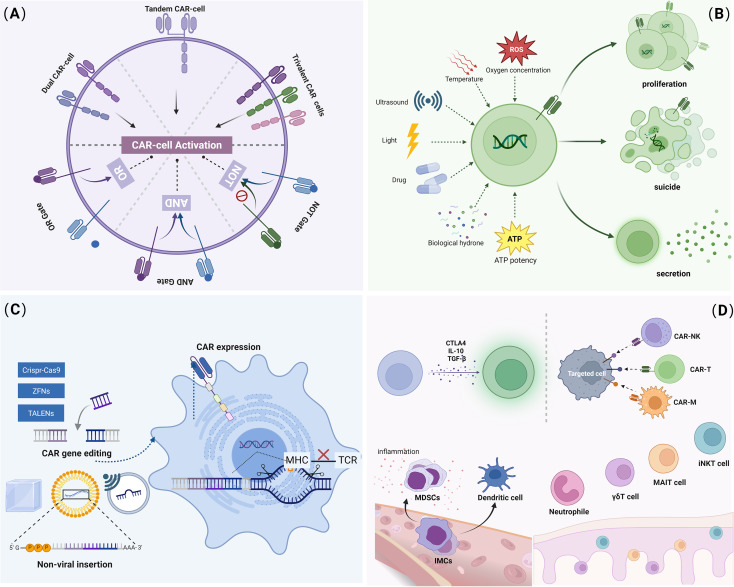
The evolving landscape of CAR engineering technology. **(A)** Enabling more efficient and controllable CAR cell activation. Multiantigen-targeting CAR T cells, including dual, tandem, and trivalent CAR T cells. Logic gate-controlled activation: ‘OR’: Activation occurs in the presence of any antigen. ‘AND’: Activation requires the simultaneous presence of two or more antigens. ‘NOT’: Activation is inhibited in the presence of a specific antigen. **(B)** Enhancing CAR cell accessibility: This process involves harvesting cells from healthy donors and utilizing nonviral gene delivery technologies, such as electroporation, microneedles, chemical carriers, and lipid nanoparticles (LNPs). It also incorporates genome editing techniques, such as CRISPR/Cas9, to disrupt T-cell receptor (TCR) and/or HLA class I molecule expression, thereby preventing graft-versus-host disease (GVHD). **(C)** Engineered immune cells beyond CAR T cells, including macrophages, NK cells, and Tregs, offer unique therapeutic advantages for treating cancer and other diseases. Future research directions include engineering other cell types, such as antigen-presenting cells, neutrophils, and stem cells, to address a broader spectrum of diseases, from chronic inflammation to acute infections. **(D)** Intelligent regulation of CAR cells: Through machine learning, multiple environmental inputs, chemical or physical signals, such as hypoxia, ATP levels, reactive oxygen species, and drugs, are integrated to ultimately determine specific functional outputs, such as cell proliferation, secretion, or programmed cell death. This process of cellular engineering transforms cell activation from traditional antigen-triggered mechanisms to multiparameter computational induction. Created at https://BioRender.com.

Therefore, the screening of candidate CAR targets should not be confined to expression profiling analysis but must be thoroughly integrated with their biological significance and pathological functions. This typically requires comprehensive evaluation of target rationality through approaches such as gene knockout models or cell depletion studies. Furthermore, to overcome the challenges posed by antigen heterogeneity and dynamic changes, therapeutic design must account for this complexity to ensure effective clearance of pathogenic cells. Currently, efforts to enhance the therapeutic performance of CAR T-cell platforms have led to multiantigen-targeting CAR designs, including dual, tandem, hybrid and trivalent CAR T cells (167). Their shared core strategy is the ‘OR’ logic gate, meaning that the presence of any one target can activate the antigen and enhance therapeutic coverage, as shown in **Figure 5A**. Dual CAR T cells express CAR molecules with two distinct binding domains, integrating signals from different targets to broaden the recognition spectrum (168). For example, in the management of refractory disorders driven by B cells or pathogenic antibodies, CD19-targeted CAR T cells have demonstrated potent B-cell depletion capabilities but limited efficacy in eliminating plasma cells. Supplementing the BCMA target partially addresses this limitation, thereby improving the therapeutic outcomes. Tandem CARs integrate two signaling domains, resulting in identical responses to different antigens and enhanced effector functions during synergistic recognition (169, 170). Hybrid and trivalent CARs can be obtained through mixed infusion or transduction of multiple CAR constructs, further expanding antigen coverage, although T-cell exhaustion must be considered (171).

Additionally, given that nonneoplastic indications require prolonged immune modulation, multiple studies have proposed strategies to optimize the CAR design, with the goal of enhancing sustainability and therapeutic efficacy (172–175). One promising approach involves engineering cell subsets, such as naive T (TN) cells and stem cell-like memory T (TSCM) cells, to proliferate into a unified phenotype with robust proliferative potential, low exhaustion characteristics, and long-term survival capacity (176, 177). Another strategy focuses on modulating the costimulatory signals that govern CAR T-cell functional persistence. CD28 promotes rapid cell activation but also predisposes cells to early exhaustion. Therefore, tuning CD28 signaling or implementing tandem costimulatory signaling domains can achieve the maintenance of a robust expansion capacity and a low-exhaustion phenotype (178–180). During design, the sequence of domains determining dominant signaling must be carefully considered.

### 4.2 Safety

During the application of CAR cells, adverse reactions may occur, necessitating a clear understanding of how safety management should be implemented. Following CAR T-cell infusion, CRS is the most prevalent toxic reaction (181). Certain clinical data suggest that CRS occurs at an extremely high frequency in patients receiving CAR T-cell therapy, with reports also emerging with respect to the treatment of autoimmune diseases (37, 39, 182–185). This effect is strongly linked to the *in vivo* proliferation of these cells and the subsequent surge in cytokines, including inflammatory mediators such as TNF-α and IFNγ, together with IL-1, IL-6, and inducible nitric oxide synthase (iNOS), which are secreted by subsequently activated monocytes and macrophages. This surge is driven by the secondary activation of multiple immune cell types rather than by the isolated action of CAR T cells (186, 187). For patients with autoimmune or inflammatory diseases, their inherent proinflammatory immune environment may further lower the threshold for systemic cytokine amplification, thereby increasing the likelihood of CRS and posing significant concerns for safety management.

Additionally, immune effector cell-associated neurotoxicity syndrome (ICANS) is recognized as a relatively frequent complication, with studies suggesting that IL-1 may mediate its development (187). More than 60% of leukemia and lymphoma patients experience this adverse event (188). Targeting normal cells or tissues that share the same antigen with the target cells is a significant concern for off-target toxicity because it can cause severe injury (189, 190). However, when CAR cell therapy is used for nonneoplastic diseases, safety considerations must extend beyond acute toxicity to focus on unique risks arising from long-term immune reconstitution, such as the clinical consequences of prolonged immune exhaustion. CAR T-cell therapies that are directed against B-cell antigens frequently induce profound depletion of the B-cell lineage and persistent hypogammaglobulinemia. This necessitates prolonged infection prophylaxis and immunoglobulin replacement therapy for patients, lasting months to years (191).

To address the aforementioned safety risks, research has focused on achieving controllable or programmable regulation of CAR T cells to manage their inherent high-risk profile. Given the possible adverse reactions that are associated with the broad cytotoxic activity of CAR T cells against multiple cell populations, CAR T-cell–based therapeutic approaches constitute a major breakthrough in achieving more precise regulation by modifying T cells to target only pathogenic cell subpopulations. Furthermore, subsequent generations of CAR T cells can persist long term in the host, with detectable populations reported for as long as a decade after infusion. For nonneoplastic diseases, which require long-term management, the risk of off-target toxicity must be urgently addressed. Accordingly, researchers are focusing on identifying modification strategies that can improve the controllability of engineered cells.

Affinity-tuned CAR cells achieve selective recognition by modulating the binding affinity of the antigen recognition domain, which enables engineered cells to maintain normal effector functions while avoiding attacks on normal cells that express physiological antigen levels (192). Additionally, as depicted in **Figure 5A**, to increase recognition specificity, a logic-gated architecture is employed to manage multiantigen signals. The ‘AND’ logic gate elevates the activation threshold by requiring that target cells simultaneously express two or more disease-associated antigens. For instance, the synNotch receptor system regulates the spatiotemporal expression sequence of two target antigens by using the initial antigen to trigger the expression of a second CAR that targets the pathogenic antigen (193). Moreover, suboptimal activation systems based on costimulatory receptors primarily modulate activation thresholds by splitting antigen activation signals and costimulatory signals between two receptors to ensure that CAR cells are activated only upon recognition of both antigens (194). The split CAR system, as a universal platform, enables ‘AND’ logic gate programming through adapter molecule design. For example, the SUPRA system pairs a CAR (zipCAR) with a free scFv (zipFv) via leucine zippers. Its application prospects extend beyond flexible antigen recognition and increasingly involve more sophisticated logic gate regulation, which requires tailored designs for different application scenarios (195). The primary construction principle of the inhibitory CAR (iCAR) incorporates ‘NOT’ logic gate techniques, which act as extra fail-safes for the suppression of unwanted immune responses. Through the use of intracellular inhibitory signaling domains, such as PD-1 or CTLA-4, the iCAR initiates an inhibitory signal that targets antigens present on non-overactive protective cell populations, thereby averting inappropriate T-cell activation (196).

Suicide-switch systems provide another level of controllability, as they facilitate the rapid elimination of CAR cells via externally activated mechanisms when uncontrolled toxicity occurs and allow CAR T cells to self-destruct. In these systems, the suicide switch is controlled to induce CAR T-cell death by a selective physical or chemical agent—for example, the presence or absence of a drug or a specific compound (e.g., iCasp9). This allows for external control and management across all stages of disease remission, aggravation, and relapse (197).

### 4.3 Accessibility

Autologous T-cell therapy eliminates the risks of GVHD and immune rejection. Its main advantages lie in its inherent compatibility, low immunogenicity, and the ability to maintain the patient-specific TCR combination, which is particularly important in cases requiring precise antigen recognition (198). However, its production remains time-consuming costly, despite viral transduction methods are still being refined (199, 200). In nonneoplastic diseases, the quality of autologous T-cells may be affected due to disease-related immune dysregulation. Chronic inflammation, long-term immunosuppressive therapy, or potential immune dysfunction can reduce the proliferation, effector function, and persistence of T-cells (201). Moreover, repeated pretreatment during the manufacturing process may lead to exhaustion or loss of T-cells, reflecting metabolic, epigenetic, and cell reprogramming disorders (202). Such limiting factors ultimately affect the expansion, phenotypic stability, and persistence of CAR T-cells, underscoring the need to address cellular biology issues and optimize process-related parameters.

Efforts to circumvent these challenges have concentrated on more accessible cellular platforms. Allogeneic CAR T cells, derived from healthy donors, have the advantages of immediate availability and more standardized quality. That means they may can achieve large-scale production, “off-the-shelf” supply, and potentially reduce costs (203). However, immunogenicity remains a critical consideration, necessitating careful donor selection or supplementary immune-modulatory strategies. Genome editing tools, such as CRISPR/Cas9, zinc-finger nucleases (ZFNs), and transcriptional activator-like effector nucleases (TALENs), and nonviral methods of transfection, including electroporation, microneedles, and chemical agent delivery, can achieve precise CAR insertion and remove or inactivate endogenous TCR or HLA molecules, thereby reducing the risk of GVHD (204). Choosing between autologous and allogeneic CAR T cells requires balancing safety, efficacy, and practicality. Autologous CAR T cells are preferred when patient-derived T cells are sufficiently healthy, avoiding GVHD is critical, or therapeutic precision depends on disease-specific T-cell characteristics, such as unique TCR diversity. Allogeneic CAR T cells may be advantageous in situations where patients have impaired or exhausted T cells, require rapid intervention, or scalable, standardized production is desired. CAR design may further optimize persistence, limit off-target effects, and allow controlled activity, particularly for nonneoplastic indications where long-term immunosuppression is undesirable. Emerging strategies such as transient mRNA expression via LNP or *in vivo* CAR engineering can further modulate these trade-offs, providing flexibility between autologous and allogeneic platforms depending on disease context. However, in certain scenarios, low endosomal escape efficiency results in insufficient effective mRNA release rates, which remains an area requiring optimization in later stages (205). Therefore, as summarized in **Figure 5B**, combining allogeneic cell harvesting, nonviral insertion, and *in vivo* or automated manufacturing represents a potential pathway to reduce costs, increase efficiency, and promote broader clinical access to CAR T-cell therapy.

### 4.4 Ethical and regulatory issues

When such potent cell therapies are applied to nonneoplastic diseases, attention to ethical and regulatory concerns regarding risk–benefit assessment, informed consent complexities, equity and cost–effectiveness is essential.

First, the criteria for evaluating risks and benefits need to be reconsidered. Unlike life-threatening cancers, chronic diseases often follow a prolonged course with complex management requirements. These conditions usually require sustained monitoring and repeated interventions, prompting a reassessment of expected benefits relative to potential risks, including symptom alleviation, long-term immunodeficiency, delayed adverse events, and healthcare system burden. Economic factors should also be incorporated into patient selection and clinical trial design to ensure that the potential benefits can outweigh the economic costs (206). Ethically, the evidence burden is greater and the criteria are more stringent when the risk is largely unquantified. In addition, there are evidentiary gaps in long-term follow-ups of chronic nonneoplastic diseases, as well as a lack of safety studies investigating such conditions. Chronic nonneoplastic diseases are still unquantified and unaddressed in relation to the aforementioned risk (3, 207, 208). This uncertainty directly affects the timing of treatment and the selection of patient populations. For instance, in infectious diseases, T cells mediate both the protection and organ pathology, and CAR T-cell therapy may cause irreversible damage to vital organs (209). In type 1 diabetes, the optimal intervention window theoretically requires early disease intervention to prevent irreversible beta-cell damage and loss (210, 211). Therefore, ethical considerations necessitate a reasonable therapeutic window and account for the risk tolerance of vulnerable populations, including children, individuals of childbearing age, and psychiatric patients. Therefore, ethical considerations require defining a reasonable treatment window, stratifying patients by disease stage, immune competence, comorbidities, and projected risk tolerance, while accounting for vulnerable populations such as children, individuals of reproductive age, and patients with psychiatric conditions.

As CAR T-cell therapy has become increasingly widespread in clinical practice, the issue of the equitable distribution of medical resources and the closely related challenge of ensuring informed consent of patients have become significant ethical concerns. Given the current imbalance between the expanding size of the potential beneficiary population and the manufacturing capacity, along with follow-up capabilities, this necessitates simultaneous consideration of disease severity, the availability of alternative therapies, uncertainties regarding long-term benefits, and biases in follow-up evidence (212–214). Informed consent in CAR cell therapy is complex because it requires patients to understand not only the treatment process and benefits but also the potential long-term immune reconstitution, risks of delayed adverse events, and limits of follow-up care and medical support, all of which must be clearly communicated by healthcare providers.

Moreover, when off-the-shelf CAR cells are considered for broader applications for non-tumor diseases, a series of additional ethical and regulatory challenges are introduced. These include but are not limited to the expanded use of donor cells, donor screening and infectious disease risk control, protection of donors’ genetic and privacy information, and the legal compliance of supply chain ethics under commercialization. Long-term immune consequences, such as persistent lymphopenia, immune dysregulation, or altered host immunity, require prospective monitoring integrated into clinical trials and post-approval surveillance.

Addressing these challenges necessitates a multilevel, dynamic, and prospective ethical governance and regulatory framework combined with adaptive clinical trial designs and strict monitoring protocols. Such a framework should encompass uniform legislation and guidelines, chronic disease-specific risk–benefit stratification frameworks, comprehensive monitoring and standardized disposal of gene products, and robust oversight of donor privacy, data security, and the commercial supply chains of allogeneic cell sources. Harmonizing these strategies with clinical translation pathways will facilitate the controlled expansion of CAR technology amid uncertainty (215, 216).

### 4.5 Cell-specific considerations

The conventional CAR-T cells are currently the most extensively studied platform. However, the engineered modifications targeting macrophages, NK cells, and Treg cells warrant further exploration for applications in nonneoplastic diseases. Given the expanding range of CAR modules and available cellular vectors, clinical practical must be tailored based on the disease context, therapeutic goals, and safety considerations, while accounting the distinct biological characteristics of each cell type.

Macrophages are highly accessible to tissues and can phagocytose pathogenic agents while reshaping the immune microenvironment. Their antigen-presenting capabilities may facilitate secondary immune responses, rendering them attractive for solid tissue pathologies requiring local immune reconstitution. However, CAR-M therapy may bring potential pro-inflammatory and pro-fibrotic consequences (217). Limited *in vivo* persistence and challenges in directing polarization toward therapeutic phenotypes remain significant hurdles. Consequently, while CAR-M holds promise for localized tissue remodeling, precise control of activation and polarization is necessary to avoid adverse effects. Given that natural killer (NK) cells have a relatively short lifespan and express inhibitory receptors, they may maintain good safety and exert protective effects compared with other cytotoxic cells. Therefore, NK cells can be used to eliminate pathogenic cells with a reduced risk of collateral damage. Owing to their short preparation timelines and flexible sources, NK cells are usable as off-the-shelf cellular products, and they are therapeutically useful and have promising potential in CAR strategies (218, 219). Most current studies have focused on acute pathologies, such as viral infections, whereas in pathological conditions in which multiple organs and systems are affected and severe inflammation is present, NK cell strategies can be used with even more protective benefits. However, more studies are needed to resolve issues such as the need to have repetitive placements and the need to address the microenvironment-determined dysfunction that can be present. These inconsistencies underscore the need for optimization strategies, such as cytokine support or microenvironment modulation, to improve durability. The innate immunosuppressive function of Tregs and their capacity for tissue homing are ideal for treating organ-specific autoimmune diseases and chronic inflammation, as they are able to restore immune homeostasis locally. Yet, phenotypic instability and variable immunosuppressive potency pose risks of inconsistent efficacy, highlighting the importance of stability-focused engineering and careful patient monitoring (220).

Most CAR-based immunotherapies to date employ a single engineered cell type. While co-administration of Tregs with CAR-T cells has been explored in oncology to reduce inflammatory toxicity without impairing effector function, the therapeutic potential of combining multiple distinct CAR-engineered cell types in nonneoplastic disease models remains unexplored (221). This gap underscores both the early stage of non-oncological CAR applications and the challenges inherent in developing multi-cellular CAR strategies. **Table 1** summarizes the principal attributes of CAR-engineered cell types, providing a reference for informed cell selection and CAR component design (222–225). Comprehensive evaluation of each engineered cell’s advantages and limitations relative to specific disease contexts is critical. Selection and optimization of CAR constructs should integrate disease-specific pathophysiology, therapeutic objectives, safety considerations, and desired duration of activity. Design elements such as costimulatory domains, trafficking signals, and cytokine support must be tailored to the selected cell type and therapeutic aim. This integrative approach facilitates rational CAR cell engineering grounded in mechanistic and clinical rationale.

**Table 1:** Comparison of different CAR cells platforms.

CAR cells	Primary mechanism of action	Therapeutic characteristics	Persistence	Safety	Accessibility	Clinical development phase	Ref.
CAR-T	cytotoxic killing cytokine release	significant therapeutic efficacy in autoimmune diseases	Long	high CRS/ICANS risk off-target toxicity long-term B-cell deficiency	self-preparation is complex, costly, and time-consuming;	clinical trial	(222)
CAR-NK	cytotoxic killing cytokine release ADCC	be applicable to acute infections	Short	low CRS/ICANS risk and MHC-independent cytotoxicity	heterogeneous preparation, diverse sources	preclinical studies	(223)
CAR-M	phagocytosis antigen presentation	demonstrates outstanding efficacy in anti-fibrosis, infection clearance, and tissue repair.	Medium	Low CRS risk, but polarity reversal risk exists	can be polarized *in vitro* or engineered *in situ in vivo*	preclinical studies	(224)
CAR-Treg	Iimmunosuppression	suitable for chronic inflammation	Medium	Low toxicity precise adjustment of the inhibition level is required.	primarily derived from autologous sources, with significant challenges in amplification and poor stability.	early clinical trials in transplantation	(225)

## 5 Conclusion and future prospects

The development of CAR technology has transformed tumor immunotherapy. More recently, this therapeutic strategy has been successfully adapted for the treatment of various nonneoplastic diseases. This success shows that CAR technology can be used across multiple pathological conditions because it provides strong immunological activity and has the ability to target tumors with great precision. The application of CAR technology for nonneoplastic diseases can be discussed from two main perspectives. One involves the modification of effector cells, including T cells, macrophages, and NK cells, to enable elimination of dysfunctional or pathological cells. This approach has been shown to effectively improve disease conditions and lead to tissue repair in autoimmune diseases, immune-associated infections, and tissue fibrosis. Another, but related, approach is immune modulation. Through this approach, modified Treg cells act to restore immune homeostasis by controlling inflammatory responses in transplant recipients and patients with chronic inflammatory conditions, such as inflammatory bowel disease and type 1 diabetes, thereby offering a clinically actionable strategy to suppress pathological immunity while preserving host defense. Importantly, the field is still transitioning from early proof-of-concept studies toward clinically mature therapeutic strategies. The current status of clinical research is presented in **Table 2**, which compiles the clinical evidence for the use of CAR cell therapies for nonneoplastic diseases and summarizes the main characteristics and the reported results of each study (226). **Table 3** presents the clinical trials of CAR technology, focusing on nonneoplastic diseases, and describes the main design characteristics of the trials.

**Table 2:** Published clinical evidence of CAR therapy for nonneoplastic diseases.

Disease	Intervention	Infusion dose	number of patients	Clinical efficacy outcome	Safety notes	Published date	Ref
SLE							
	CD19 CAR T cell	1×10^6^/kg	1	Achieve a rapid decline in autoantibody levels and improvement in disease activity scores within one month.	No CRS or ICANS	2021 Aug	(36)
	CD19 CAR T cell	1×10^6^/kg	5	All patients achieved disease remission criteria within 3 months and maintained long-term relapse-free status following B-cell reconstitution (approximately 110 days on average)	Grade 1 CRS in 3 of 5 patients; no ICANS	2022 Oct	(37)
	BCMA-CD19 CAR T cell	1.5-3×10^6^/kg	12	All patients achieved 100% remission and maintained drug-free recovery.	Grade 1 CRS; no ICANS	2023 Jun	(52)
	allogeneic CD19 CAR-T cell	1×10^6^/kg	4	All patients demonstrated significant improvement in clinical symptoms and signs, with sustained declines in antibody levels. One patient achieved drug-free remission.	Grade 1 CRS; no ICANS or GVHD	2025 Aug	(55)
SSc							
	CD19 CAR T cell	1×10^6^/kg	1	Rapid improvement in cardiac, joint, and skin manifestations with successful seroconversion	Grade 1 CRS; no ICANS	2023 Aug	(39)
	CD19 CAR T cell	5×10^6^/kg	1	Sustained improvement in lung function, marked reversal of radiographic findings, and a persistent decline in autoantibody levels lasting up to 11 months.	Grade 1 CRS; no ICANS	2024 Mar	(41)
	allogeneic CD19 CAR-T cell	1×10^6^/kg	3	All patients achieved deep remission, shown by the significant improvement in the clinical response index scores and the reversal of inflammation and fibrosis.	No CRS or ICANS	2024 Sep	(54)
	CD19 CAR T cell	1×10^6^/kg	6	Significant improvement in skin and lung fibrosis was observed, and disease progression was halted during a median follow-up period of 487 days.	Grade 1 CRS in 3 of 6 patients; Grade 2 CRS in 2 of 6 patients; no ICANS	2025 Feb	(42)
SLE(8 patients), IIM(3 patients)and SSc (4 patients)							
	CD19 CAR T cell	1×10^6^/kg	15	All patients achieved drug-free clinical remission with disappearance of autoantibodies, and no severe toxicity was observed.	Grade 1 CRS in 10 of 15 patients; Grade 2 CRS in 1 of 15 patients; One patient mild ICANS; One patient had grade 4 neutropenia	2024 Feb	(38)
RA							
	CD19 CAR T cell	1×10^6^/kg	1	The patient’s condition rapidly improved, with simultaneous sharp declines in RF, ACPA, and CRP levels.	severe CRS and ICANS	2025 Feb	(43)
Myasthenia gravis							
	BCMA CAR T cell	Dose level 1:3·5 × 10^6^/kg Dose level 2:17·5 × 10^6^/kg Dose level 3: 52·5 × 10^6^/kg	14	Clinical symptoms have improved, but no significant decrease in autoantibodies has been observed.	No CRS or ICANS	2023 Jul	(44)
	BCMA CAR T cell	1×10^8^/kg	1	Significant reduction in autoantibody levels and improvement in muscle strength and fatigue	No CRS or ICANS	2023 Dec	(45)
ITP							
	CD19 CAR T cell	0.5×10^6^/kg	1	The patient’s platelet count successfully rebounded and reduced the level of autoantibodies.	Grade 1 CRS;no ICANS	2024 Jul	(49)
	CD19 CAR T cell	1×10^8^/kg	1	The patient’s platelet count rapidly returned to normal and remained stable, while simultaneously achieving clearance of the pathogenic autoantibodies.	Grade 1 CRS;no ICANS	2025 Jan	(50)
AIHA							
	CD19 CAR T cell	0.5×10^6^ cells/kg	8	All patients achieved complete remission and long-term drug-free remission.	Grade 1 CRS in 6 of 8 patients; Grade 2 CRS in 2 of 8 patients; Grade 1 ICANS in 1 of 8 patients	2024 Nov	(51)
UC							
	CD19 CAR T cell	1×10^6^/kg	1	Decreased antibody levels and sustained clinical and biochemical remission	Grade 1 CRS;no ICANS	2025 Sep	(226)

SLE, Systemic lupus erythematosus; SSc, Systemic sclerosis; IIM, Idiopathic inflammatory myositis; RA, Rheumatoid arthritis; MG, Myasthenia gravis; ITP, Primary immune thrombocytopenia; AIHA, Autoimmune hemolytic anemia; UC, Ulcerative colitis.

**Table 3:** Open ongoing clinical trials of CAR cell therapy for nonneoplastic diseases.

Disease	Clinical trial number	Target	Cell source	Enrollment	Phase	Status	Primary outcome measures
SLE	NCT06150651	CD19	T cell	6(Estimated)	I	Recruiting	DLT and AEs
	NCT06585514	CD19	T cell	18(Estimated)	I/II	Recruiting	Phase I:DLT and AEs Phase II: ORR
	NCT05938725	CD19	T cell	6	I/II	Active, not recruiting	Phase I:DLT and AEs Phase II: CRR
	NCT05846347	CD19-BCMA	T cell	15(Estimated)	I	Unknown	DLT and AEs
	NCT06839976	CD19	T cell	24(Estimated)	I/II	Recruiting	DLT
	NCT07233642	CD19	T cell	18(Estimated)	I	Recruiting	AEs and disease activity index
	NCT06121297	CD19	T cell	28(Estimated)	I/II	Recruiting	AEs
SSc	NCT06328777	CD19	T cell	12(Estimated)	I/II	Recruiting	AEs
MG	NCT06371040	CD19-BCMA	T cell	9(Estimated)	I	Recruiting	AEs
	NCT05451212	MuSK	T cell	7	I	Completed	DLT and AEs
	NCT06359041	CD19	T cell	12(Estimated)	I/II	Recruiting	AEs
	NCT06193889	CD19	T cell	66(Estimated)	II/III	Recruiting	AEs and efficacy
RA	NCT06475495	CD19	T cell	13(Estimated)	I/II	Not yet recruiting	AEs
	NCT07100873	CD20	γδ T cell	25(Estimated)	I	Recruiting	DLT
	NCT06613490	CD19	NK cell	24(Estimated)	Early I	Recruiting	DLT and AEs
ITP	NCT05315778	BCMA	T cell	5(Estimated)	II	Unknown	overall response including CR and PR
	NCT06519565	BCMA	T cell	6(Estimated)	Early I	recruiting	AEs and the safe dosage for a single infusion
	NCT06337474	CD19	NK cell	9(Estimated)	Early I	recruiting	DLT and AEs
	NCT06787989	CD19-BCMA	T cell	20(Estimated)	I	recruiting	AEs
	NCT06352281	Unknown	T cell	10(Estimated)	I/II	recruiting	Clinical response
AIHA	NCT06212154	CD19	T cell	13(Estimated)	I	recruiting	MTD and AEs
	NCT06920446	CD19-BCMA	Universal T cell	18(Estimated)	Early I	Not yet recruiting	DLTs and TEAEs, Clinical response rates
HIV infection	NCT03240328	CD4 binding site on gp-120	T cell	40(Estimated)	I	recruiting	AEs
	NCT03617198	CD4 binding site on gp-120	T cell	12	I	active, not recruiting	AEs
HIV infection	NCT04863066	CD4 binding site on gp-120	T cell	8(Estimated)	I	unknown status	AEs
	NCT04648046	CD4 binding site on gp-120	T cell	15(Estimated)	Early I	recruiting	DLT and AEs
Kidney Transplant	NCT04817774	HLA-A2	Treg	26	I/II	Completed/Follow-up ongoing (see NCT05987527)	TEAEs
	NCT05987527	HLA-A2	Treg	11(Estimated)	Unknown	active, not recruiting	AEs
Liver Transplant	NCT05234190	HLA-A2	Treg	33(Estimated)	I/II	recruiting	DLT and TEAEs
GVHD	NCT05993611	CD6	Treg	27(Estimated)	I	Suspended	DLT and Feasibility
CD	NCT05239702	CD7	T cell	75(Estimated)	Early I	Unknown	DLT and AEs
CD and UC	NCT07309744	CD19-BCMA	T cell	30(Estimated)	Early I	Not yet recruiting	AEs
Type 1 Diabetes	NCT07142161	CD7	T cell	3(Estimated)	Early I	active, not recruiting	DLT and AEs

SLE, Systemic lupus erythematosus; SSc, Systemic sclerosis; MG, Myasthenia gravis; RA, Rheumatoid arthritis; ITP, Primary immune thrombocytopenia; AIHA, Autoimmune hemolytic anemia; GVHD, Graft-versus-host disease; CD, Crohn’s disease; UC, Ulcerative colitis; DLT, dose-limiting toxicity; AEs, adverse events; SAEs, serious adverse events; TEAEs, treatment-emergent adverse events; ORR, objective response rate; CRR, complete response rate; CR, complete response; PR, Partial response; MTD, maximum tolerated dose.

Overall, CAR technology holds promise for achieving dual immune regulation through both precisely targeted cellular elimination and remodeling of the immune microenvironment, offering a novel therapeutic paradigm for diseases that remain difficult to control with conventional approaches. Clues for possible new therapeutic methodologies and future clinical advancements across various conditions may also be garnered through the successful investigation of the use of CAR cell therapy for these disorders. Current discussions regarding adverse clinical outcomes, incomplete follow-up evidence, and underlying biological risks remain relatively limited. While advancing foundational innovations in the use of engineered cells, efficacy evaluations must rely on multicenter, long-term, large-scale follow-up clinical trials to facilitate the actual clinical transformation and implementation of CAR technology into nononcological applications.

Future CAR technology is expanding in multiple dimensions. (i) We anticipate that the scope of future cell engineering will extend to other immune cell populations beyond conventional CAR T cells, encompassing a broader immune ecosystem, as illustrated in **Figure 5C**. For example, APCs mediate T-cell killing through their antigen uptake and presentation functions. Modifying APCs at the source holds promise for regulating ‘immune education’. Engineered tolerogenic APCs can collaborate with Tregs to establish immune tolerance both upstream and downstream. A previous study demonstrated that IL-10 gene transfer via a lentiviral vector led to successful generation of tolerogenic dendritic cells (tolDCs), which were capable of stably inducing Treg differentiation (227). Furthermore, chimeric antigen receptor dendritic cells (CAR DCs) with targeting capabilities have demonstrated therapeutic potential in preclinical models by enabling engineered APCs to directly participate in immune regulation (228). Owing to their rapid mobilization and potent chemotaxis, neutrophils are considered for clearing acute infections or refractory biofilms. However, the *in vivo* activity of neutrophils remains constrained by their inherently short lifespan and limited sustained functionality (229, 230). To transform this natural advantage of chemotaxis into a controllable therapeutic strategy, one proposed engineering strategy involves the introduction of CAR constructs into human pluripotent stem cells (hPSCs), followed by their directed differentiation into neutrophils. Each of these aspects addresses a different, but important, component of transduction efficacy and supply. Engineered neutrophils have applications beyond tumor environments and could be useful in infection scenarios. Via CAR-mediated targeting, these cells can penetrate sites of infection or biofilm enclaves and release intracellular bactericidal granules that are able to exert antimicrobial action beyond the reach of standard antibiotics (231). Other unconventional lymphocyte subsets are also of interest. In particular, γδT cells, mucosa-associated invariant T (MAIT) cells, and type I natural killer T (iNKT) cells have garnered attention because of their innate gut and liver homing characteristics and because they may be able to perform particular functions in metabolic and inflammatory diseases. The mechanisms that lead to the long-term persistence of these innate-type T cells and their compatibility with CAR technology are poorly understood (232, 233). The strong immunosuppression exerted by myeloid-derived suppressor cells (MDSCs) may offer the potential for the creation of tolerogenic microenvironments. However, their possible tumor-promoting effects in chronic disease situations need to be considered carefully (234). Mesenchymal stem cells also provide a unique opportunity. Their self-renewal and multilineage differentiation potential indicate that they can be considered living drug factories that can home to injured tissues (235). Future designer cells will be engineered to incorporate more rational modifications on the basis of an understanding of these unique cellular characteristics, thereby enabling the creation of the first responsive and controllable living therapeutics for complex disease environments.

(ii) Furthermore, advances in CAR technology hinge on interdisciplinary convergence. One highly promising frontier involves integration with nuclear medicine to develop a novel radioimmunotherapy–synergistic integrated diagnosis and treatment model. On the one hand, the use of immune PET tracers enables real-time, noninvasive, and quantitative dynamic monitoring of the *in vivo* distribution of CAR T cells, which holds great promise for optimizing clinical decisions. Through direct cell labelling or more sophisticated reporter gene imaging techniques, as well as immune PET technology targeting endogenous immunomarkers, it is possible to track the systemic biodistribution and migration dynamics of engineered target cells, thereby providing a strategy for evaluating the efficacy of immunotherapies (236–238). On the other hand, an emerging concept involves the transformation of CAR T cells into ‘biological missiles’ for targeted radionuclide delivery. Genetically engineered ‘Thor’ cells enable highly sensitive tracking of radionuclides via PET imaging while precisely delivering lethal alpha-emitting radionuclides (239). Moreover, in oncology, drug delivery strategies that are based on ultrasound-mediated microbubble destruction may offer a promising platform for achieving CAR gene loading and localized delivery (240). Other media, such as silica nanoparticles, biomimetic coatings, and hydrogels, have immense potential as delivery vehicles. Simultaneously, collaboration in the field of artificial intelligence holds promise for designing more complex perception–feedback–execution systems. Integrating AI into the CAR design goes beyond structural enhancements into the realm of smart circuitry. In **Figure 5D**, engineered cells can be viewed as programmable cell decision units. In this case, molecular biology goes hand in hand with machine learning to predict critical combinations of signals and, in turn, fabricate perception modules and synthetic gene circuits. This creates ‘intelligent’ engineered cells that can detect different signals of the immune microenvironment, such as the temperature, hypoxia, an increased ATP level, or specific signaling factors. Moreover, engineered cells can respond by proliferating, secreting specific factors, or undergoing apoptosis (241–243). Beyond these innovations, combinatorial approaches are particularly relevant in non-neoplastic diseases. Combined with immunomodulatory drugs, biologics, antiviral drugs, anti-fibrotic drugs, etc., it may be a necessary strategy to achieve the expected therapeutic effect (244–246). The aforementioned interdisciplinary technologies may enable non-invasive determination of the optimal usage time and sequence of each drug, as well as autonomously generating combined loads based on local signals. This means it can optimize the combined treatment plan, precisely regulate the immune response, and maximize the therapeutic effect of CAR cells. It is our belief that CAR therapy will evolve from a single-acting active drug to the core regulator of personalized and multimodal treatment strategies, and the innovative integration of multiple fields of research will continue to improve the design of smarter, more accurate, and more accessible models of immunotherapy and will open new avenues for the treatment of previously intractable diseases.

## Glossary

CAR: chimeric antigen receptor
TCR: T-cell receptor
MHC: major histocompatibility complex
ALL: acute lymphoblastic leukemia
MM: multiple myeloma
DLBCL: diffuse large B-cell lymphoma
FL: follicular lymphoma
SLE: systemic lupus erythematosus
RA: rheumatoid arthritis
MG: myasthenia gravis
CRS: cytokine release syndrome
AIDS: acquired immunodeficiency syndrome
HIV: human immunodeficiency virus
iC9: inducible cysteine protease 9
HSV-TK: herpes simplex virus thymidine kinase
FAP: fibroblast activation protein
LNP: lipid nanoparticles
MI: myocardial infarction
SASP: senescence-associated secretory phenotype
uPAR: urokinase-type plasminogen activator receptor
NK cell: natural killer cell
GVHD: graft-versus-host disease
HSCT: hematopoietic stem cell transplantation
IBD: inflammatory bowel disease
Tregs: regulatory T cells
nTregs: natural regulatory T cells
UC: ulcerative colitis
T1DM: type 1 diabetes mellitus
CTL: cytotoxic T lymphocyte
TNF: tumor necrosis factor
iNOS: inducible nitric oxide synthase
ICANS: immune effector cell-associated neurotoxicity syndrome
iCAR: inhibitory CAR
ZFNs: zinc-finger nucleases
TALENs: transcription activator-like effector nucleases
APCs: antigen-presenting cells
hPSCs: human pluripotent stem cells
MAIT: mucosa-associated invariant T cells
MDSCs: myeloid-derived suppressor cells
